# Far from Naturalness: How Much Does Spatial Ecological Structure of European Tree Assemblages Depart from Potential Natural Vegetation?

**DOI:** 10.1371/journal.pone.0165178

**Published:** 2016-12-22

**Authors:** Giovanni Strona, Achille Mauri, Joseph A. Veech, Günther Seufert, Jesus San-Miguel Ayanz, Simone Fattorini

**Affiliations:** 1 European Commission, Joint Research Centre, Directorate D - Sustainable Resources – Bio-Economy Unit, Ispra (VA), Italy; 2 Department of Biology, Texas State University, San Marcos, United States of America; 3 Department of Life, Health & Environmental Sciences, University of L’Aquila, L’Aquila, Italy; 4 CE3C – Centre for Ecology, Evolution and Environmental Changes / Azorean Biodiversity Group and Universidade dos Açores - Departamento de Ciências Agrárias, Angra do Heroísmo, Açores, Portugal; Aristotle University of Thessaloniki, GREECE

## Abstract

Contemporaneous plant communities may retain a mark of past disturbances in their ecological patterns. However, unraveling the history of disturbance on natural systems at a large scale is often unfeasible, due to the complexity of the factors involved and lack of historical data. Here we aim at demonstrating how comparing observed spatial structure of tree assemblages with that expected in a hypothetical, undisturbed scenario can shed light on how natural European forests are. Borrowing an analytical approach developed in the field of network analysis, we assessed how much the observed ecological patterns of nestedness (i.e. positive co-occurrence), segregation (i.e. negative co-occurrence), and modularity in tree assemblages deviate from randomness, and from those projected by Potential Natural Vegetation (PNV) geobotanical expert assessments. We found clear evidence that European forests are far from a natural condition, showing only moderate signals (especially at higher latitudes) of the ecological spatial structure typical of undisturbed vegetation (i.e. nestedness). Our results highlight how taking into account spatial structure along with diversity can be a fundamental tool to address this problem and assess the degree of naturalness in species assemblages.

## Introduction

Patterns in species spatial arrangements, such as, for example, the tendency for species occurrences to overlap or segregate, can emerge from a variety of ecological processes related to species interactions, habitat filtering, dispersal, colonization and extinction [1–4]. These processes usually work on a long-term temporal scale, which means that structural patterns will emerge slowly and under the condition that the structuring forces remain steady. Conversely, cycles of deforestation, abandonment, and afforestation, as well as natural disasters such as fires and storms, may create, in the most disturbed areas, scenarios where the remnants of the original natural vegetation are hardly detectable.

In a simplified view, this would suggest that undisturbed vegetation should be highly and consistently structured, and that, conversely, different types of disturbance would reduce the strength of structural patterns at landscape level. At local level, a particular disturbance may act as an environmental filter limiting diversity and allowing only few species to persist with very little variation in space (e.g. overgrazing). But, at the landscape level, where typically different types of disturbance operate simultaneously, the overall effect would be a loss of structure.

Due to the variety and complexity of factors responsible for spatial structure in local species assemblages [5–8], identifying likely scenarios expected in absence of disturbance is not straightforward. In this context, expert-based maps of Potential Natural Vegetation (PNV) could offer a possible key of interpretation.

The concept of PNV has been defined in several ways [9]. Here we refer to PNVs as the plant communities that would become established in given areas if all successional sequences were completed under the present local climatic and edaphic conditions without interference by humans [10–12]. Maps of PNV have been produced mostly as a tool to orient conservation strategy [13]. Nevertheless, they could also constitute a valuable source of baseline information to evaluate qualitative and quantitative discrepancies between the observed and the expected structure of plant communities.

Among the various forms of spatial structure, ecologists have been most often interested in investigating species co-occurrence, i.e. in assessing if (and to what extent) the presence of a particular species in a local community is positively or negatively affected by the presence/absence of other species [14–16]. Although, at a local scale, competition can lead to negative co-occurrence between species having similar functional traits/niches, at a larger scale, environmental filtering would promote spatial consistency in associations of species having similar tolerance to abiotic factors and complementary functional traits (e.g. mature vegetation types), and resulting in patterns of positive co-occurrence [17–21]. Of course, resource availability, and hence local diversity, can modulate these patterns, with competitive exclusion being more pronounced in areas with moderate to high diversity [22] than in extreme environments or isolated habitats [23].

The general tendency for species co-occurence can be assimilated to the concept of nestedness, which is a pattern where any species assemblage tends to be a subsample of richer assemblages, and which can be promoted by differential extinction, dispersion and colonization [24]. There is no consensus on whether or not a gradient in species richness among sites (as well as a gradient of variation in species abundance among sites) is a necessary condition for nestedness [24–26]. In our reasoning, however, the consistency of species association can be considered a mark of ecosystem maturity (and hence stability) regardless of differences in species diversity. Thus, we will stick to the concept of nestedness as in [25, 26], considering nestedness simply as a measure of overlap in species composition per site and in species site occurrence. Instead, we will refer to the opposite tendency for non-overlap as segregation, again as in [26].

Another interesting pattern that is becoming more and more popular is modularity [27], which occurs when clusters of similar species composition can be identified among species assemblages [28]. Modularity can originate from discontinuous (and synchronous) responses of species to environmental gradients, when differences in resource or habitat specialization among species—and environmental heterogeneity—promote species repartition among different, spatially separated clusters [27]. Although a better understanding of patterns and processes associated with modularity is of great biogeographical interest, the study of modularity has received little attention, probably because of the lack of appropriate methodological tools [29, 30]. Only recently, the increasing incorporation of network theory into ecological studies [31] has opened the way.

Understanding the processes that can lead to nestedness, segregation, and modularity is a complex task. Nevertheless, it is intuitive that any structural pattern emerging from natural ecological processes would become more and more evident as these remain active without interruption. At any time, external disturbances could stop such processes and thus modify the structural patterns built up to that moment. Therefore, it should be possible to quantify the exposure to disturbance of a given species assemblage as a function of its spatial structure or lack thereof.

Sources of disturbance could be either natural phenomena (such as fires, storms, earthquakes, landslides, epidemic diseases [32]), and human activities. However, the first kind of processes is most often limited to a local scale, making anthropic influence the main shaper of large scale patterns. Furthermore, ‘natural’ disasters, despite their unpredictability and stochasticity, are frequently due to human activities [33]. Unwise management often creates risky situations that make the happening of certain dramatic events more likely. For example, growing exotic trees may increase the risk of fires [34], and/or the risk of epidemics due to moving alien pathogens [35]. The risk of epidemics may be severely increased also by reducing local diversity, by selective growing [36, 37], as well as deforestation may increase the risk of landslides and floods [38].

Landscapes in Europe have been modified for millennia through clearance of forests to create croplands and pastures, and by intensive tree collection for fuel wood and construction materials [39]. Although the industrial revolution has certainly played a major role in this process, many European regions underwent profound deforestation thousands of years before that period and several areas have been altered since the mid-Holocene due to the establishment of the first European agricultural societies [40]. As a consequence, there are almost no intact forest landscapes left in Europe, with the exception of small patches in Fennoscandia and North Eastern territories [41]. Thus, we should expect to find much evidence of past disturbances in the current spatial structure of tree assemblages.

Quite surprisingly, only a few studies have focused on this topic. Moreover, these have been conducted only at a regional scale, and gave contrasting explanations to the observed nestedness patterns (see, for example, [42, 43]). Here we try to fill in this gap by (i) developing an innovative approach to investigate ecological spatial structure, and (ii) by applying it to the most comprehensive dataset of tree species available for Europe [44], which includes almost one million occurrences. In doing this, we show that comparing the spatial structural patterns observed for actual vegetation (ACV) with those predicted by PNV assessment can provide valuable insight as to how much contemporaneous tree assemblages depart from naturalness.

## Materials and Methods

### Use of PNV as a benchmark

The concept of PNV has been the focus of much debate regarding both its formal definition and meaning (see [45] for a review). In particular, it has often been questioned whether it is reasonable to use PNV as a qualitative benchmark to evaluate the status of actual vegetation (ACV) [9]. We agree that multiple factors can potentially affect species composition and diversity of the vegetation observed in a given area in a very site-specific way, so that PNV is not an exact representation of natural mature vegetation. Due to this, some researchers have questioned whether the concept of PNV is useful in any way [9]. Despite this concern, we put aside the conceptual problems of defining exact PNVs, and we built on the idea that the communities depicted by PNVs, in principle, should conform to one among the various ‘reasonable’ ecological outcomes for a given area. Here, the term ‘reasonable’ indicates compatible with the local environmental features. Thus, even without assuming that the prediction of PNV represents the exact mature vegetation (in terms of species composition) that would emerge under the assumption of environmental stability and in the absence of human disturbance [11], it is appropriate to consider a particular PNV as the best available description of the plausible vegetation of an area [12, 45, 46] in terms of ecological structure.

### Tree species data

Actual tree distribution was obtained from Euforest, a new tree occurrence dataset provided by the European Commission [44], which includes almost 1 million occurrence data for 242 tree species across all Europe. Euforest combines the information from three large datasets provided by the European Forest Data Centre of the European Commission (http://efdac.jrc.ec.europa.eu) and particularly: (1) presence-absence data from 22 National Forest Inventories, (2) plot information from the so-called Level I and Level II schemes set up by countries for the monitoring of atmospheric pollution on forests in the context of Regulation No. 2152/2003 (Forest Focus, [47]), and (3) the Biosoil project [48], in which forest tree biodiversity was sampled in 3,379 plots in Europe. All data were aggregated at a spatial accuracy of 1 square km, by assigning plot presences to the cell centroids of an INSPIRE compliant 1 km × 1 km grid, specifically designed for pan-European mapping (see [44] for details).

Then, we generated a dataset mapping the potential occurrences (PNV) using the map of Bohn et al. [49]. We included in the study only the 190 tree species present in both Bohn et al.’s map and Euforest. We assembled the PNV dataset by projecting the positions of the Euforest plots on the map of Bohn et al. [49], which consists of large numbers of polygons, each associated with a certain vegetation type that, in turn, corresponds to a particular set of plant species. For each plot location, we evaluated the presence/absence of the 190 tree species from the set of species associated with the corresponding polygon. In this way, we obtained two comparable datasets. In order to make clear that we used only tree species in our analyses we will henceforth use the abbreviations ACV_T_ for the actual tree vegetation, and PNV_T_ for the potential tree vegetation.

Because our analysis focuses on tree species interactions, we limited our study to forested areas, in order to exclude localities (such as shrublands) where trees, even if listed in our dataset, are likely to occur at densities too low for species interaction to be a structuring process. For this, we filtered both ACV_T_ and PNV_T_ datasets using the global land cover classification by [50], in order to include only those located in areas categorized as (1) evergreen needleleaf forest; (2) evergreen broadleaf forest; (3) deciduous needleleaf forest; (4) deciduous broadleaf forest; and (5) mixed forest and woodland.

**Fig 1:**
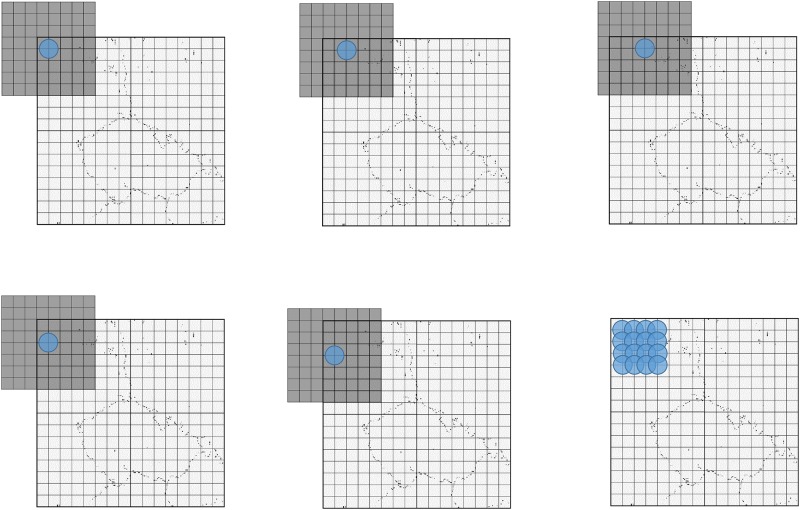
Explanation of the moving window approach used to build species/area matrices and to geo-reference structural values. The window consisted of a regular square grid of 100 cells (each having size 0.1° latitude × 0.1° longitude). The window was moved across all of Europe by displacements of 0.1° latitude or longitude at a time. At each repositioning, the window was superimposed on both ACV_T_ and PNV_T_ distribution maps in order to create two species × site matrices.

To investigate spatial structure we used a moving window [51] consisting of a regular 10 × 10 grid having size 1° latitude × 1° longitude, with each grid cell being 0.1° × 0.1°. The grid was moved across all of Europe by displacements of 0.1° latitude or longitude at a time (Fig 1). At each repositioning, the grid was superimposed on both ACV_T_ and PNV_T_ distribution maps (i.e. the two tree distribution maps based, respectively, on the three above mentioned datasets, and on the map of PNV [49]) in order to create two species × site matrices including the presence-absence of each tree species of the dataset in each one of the grid cells (n = 100 “sites”) according to, respectively, ACV_T_ and PNV_T_ distribution maps. Only non-empty columns (i.e. grid cells–i.e. “sites”—hosting at least one tree species) and non-empty rows (i.e. tree species found in at least one grid cell) were included in each resulting matrix. To ensure robustness of results, only matrices including at least 5 columns (i.e. “sites”) and 5 rows (i.e. tree species) were retained (leading to a total of 34,353 matrices).

Each structural measure computed on a given matrix (corresponding to a 1° latitude × 1° longitude area) was then assigned to the centroid of the corresponding window. In this way, we obtained information at a spatial resolution of 0.1 × 0.1 latitudinal per longitudinal degrees.

### Quantifying ecological structure

We quantified community spatial structure in both ACV_T_ and PNV_T_ assemblages by using the $\overset{-}{Ɲ}$ statistic [26]. The $\overset{-}{Ɲ}$ metric aims at assessing whether species in ecological networks and food webs tend to share interacting partners/resources more (or less) than random expectation. The metric is computed as the average, normalized deviation between the observed and the expected number of partners shared by any pair of nodes. Although first conceived for network analysis, this measure can be easily applied to species presence-absence matrices since, in principle, these correspond to bipartite networks, i.e. networks where nodes can be divided into two disjoint sets, one having only in-coming links (e.g. localities inhabited by some species), and the other one having only out-going links (e.g. species inhabiting some localities). The applicability of structural measures conceived for community matrix analysis to bipartite networks (and *vice versa*) has been widely investigated in studies dealing with nestedness [52], species co-occurrence [53], and modularity [27].

The $\overset{-}{Ɲ}$ measure makes it possible to investigate, in a single analytical step, patterns of nestedness, segregation (here meant as the tendency of a species/area matrix towards ‘checkerboardness’, as a potential result of competitive exclusion [14]) and modularity.

The general formula to compute the measure for a pair of nodes *V*_*i*_ and *V*_*j*_ (i.e. species or localities) is:

$${Ɲ}_{ij}=\frac{\left(S_{ij}-P_{ij}\right)}{min(d_{i},d_{j})}\times \frac{1}{\Omega_{ij}}$$ (1)

Where *S*_*ij*_ is the actual number of neighbors shared by *V*_*i*_ and *V*_*j*_ (for example, the number of localities where both species *i* and *j* occur, when computing species overlap; or the number of species occurring at both locality i and j, when computing site overlap). *P*_*ij*_ is the expected number of shared neighbors, *d*_*i*_ and *d*_*j*_ are the respective node degrees (i.e. the number of localities where species *i* and *j* occur respectively, or the number of species respectively found at locality *i* and *j*). *Ω*_*ij*_ is a standardization parameter corresponding to the maximum theoretical value of Ɲ_*ij*_ (see [26] for details). $\overset{-}{Ɲ}$ is then computed as the average of all Ɲ_*ij*_ pairs, while the standard deviation of Ɲ_*ij*_ values provides a measure of modularity.

The key point of the method is the application of a probabilistic approach to compute the expected number of shared nodes:

$$P_{ij}=\sum_{k=1}^{\text{min}\left(d_{i},d_{j}\right)}\frac{\left(\begin{array}{c} n\\ k \end{array}\right)\times \;\left(\begin{array}{c} n-k\\ d_{j}-k \end{array}\right)\times \;\left(\begin{array}{c} n-d_{j}\\ d_{i}-k \end{array}\right)}{\left(\begin{array}{c} n\\ d_{j} \end{array}\right)\times \;\left(\begin{array}{c} n\\ d_{i} \end{array}\right)}\times k$$ (2)

The $\overset{-}{Ɲ}$ metric can vary between -1 (indicating complete spatial segregation) and 1 (indicating perfect nestedness).

For each matrix, we computed both $\overset{-}{Ɲ}$ and modularity. In the context of our analysis, Ɲ_ij_ values represent the degree of overlap in distribution between two tree species, or the degree of overlap in species composition between two localities (i.e. grid cells). We assessed significance of $\overset{-}{Ɲ}$ values using a *Z* test [26].

### Comparing the structure of ACV to PNV

The difference in ecological structure between ACV_T_ and PNV_T_ provided by $\overset{-}{Ɲ}$, was mapped at a resolution of 0.1 latitudinal × longitudinal degrees. In addition, to investigate $\overset{-}{Ɲ}$ changes for regions with more homogeneous environmental conditions, species communities and ecological processes, we averaged the statistics per eco-region [54] and constructed ecoregion-based maps of the differences between $\overset{-}{Ɲ}$ of ACV_T_ and $\overset{-}{Ɲ}$ of PNV_T_. The use of ecoregions has also been suggested as an informative approach for biodiversity conservation assessment [55].

### Intact forest landscapes in Europe

Europe is extremely poor in terms of intact forest landscapes (IFL) [41]. Moreover, some of the most important old-growth forests (such as the Białowieża Forest in Poland [56]) are not covered by the Euforest dataset [41, 44]. Nevertheless, there is some IFLs for which the Euforest dataset provides tree occurrence data. We used those areas (that we identified by filtering the Euforest dataset using the global IFL map for the year 2000, available at http://www.intactforests.org/index.html) to challenge our starting hypothesis, i.e. to verify if their tree assemblages showed, as expected, a high degree of structure resulting from the absence of disturbance.

### Sensitivity analysis

Although the data we have used to define actual tree vegetation (the Euforest dataset, [44]) represent the best information available on European tree distribution, as for any empirical dataset, we cannot consider this information as complete. Moreover, the fact that data were collected at national level, could possibly lead to a heterogeneous distribution of biases across Europe. We investigated whether these potential issues affected our results and conclusions by performing a robust sensitivity analysis. Since the data about PNV can be considered virtually complete, we focused on ACV, replicating the structural analyses (i.e. the computation of nestedness, segregation and modularity) by removing at random 50% of occurrences from the Euforest dataset, and then comparing these results with those obtained using the complete dataset.

## Results

**Fig 2:**
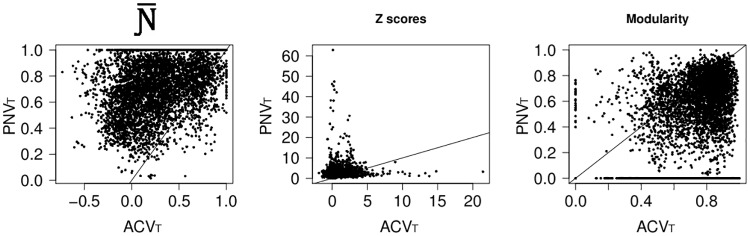
Difference in spatial ecological structure between ACV_T_ and PNV_T_. Relationships between $\overset{-}{Ɲ}$, Z-score and modularity values computed for ACV_T_ and the corresponding values computed for PNV_T_ in all 1° latitude × 1° longitude matrices. Diagonal lines of equality provide a visual guide for seeing whether ACV_T_ or PNV_T_ had the greater value.

**Fig 3:**
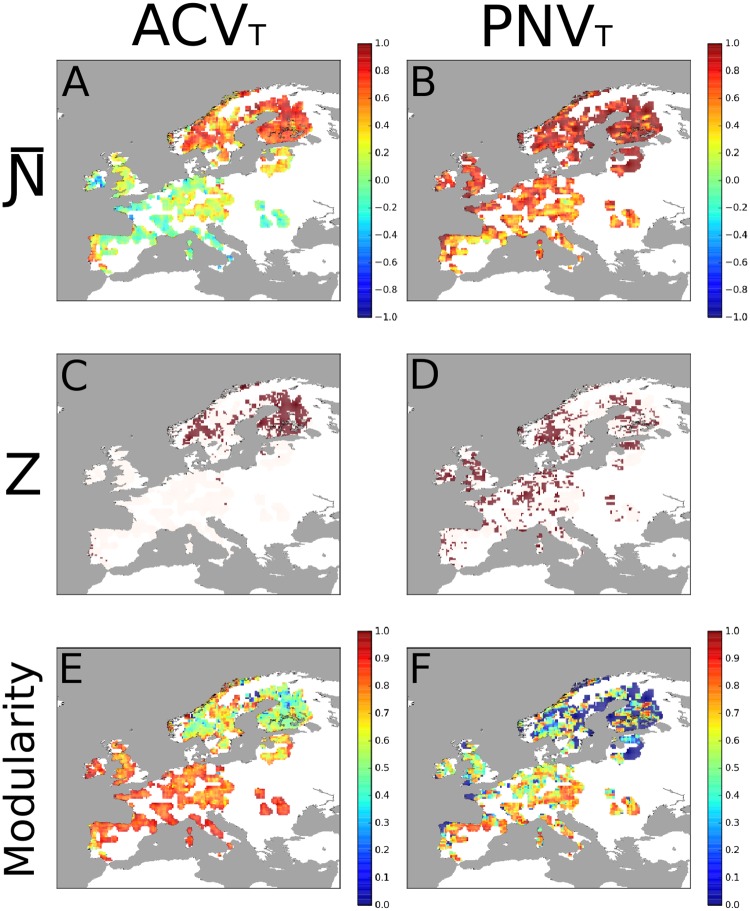
Mapping the structure of ACV_T_ and PNV_T_ at 1° latitude × 1° longitude resolution. $\overset{-}{Ɲ}$, Z and modularity values computed in all 1° latitude × 1° longitude matrices for both ACV_T_ and PNV_T_. Red pixels in mid panels correspond to matrices having Z>2, which indicates significance of $\overset{-}{Ɲ}$ at p<0.05.

**Fig 4:**
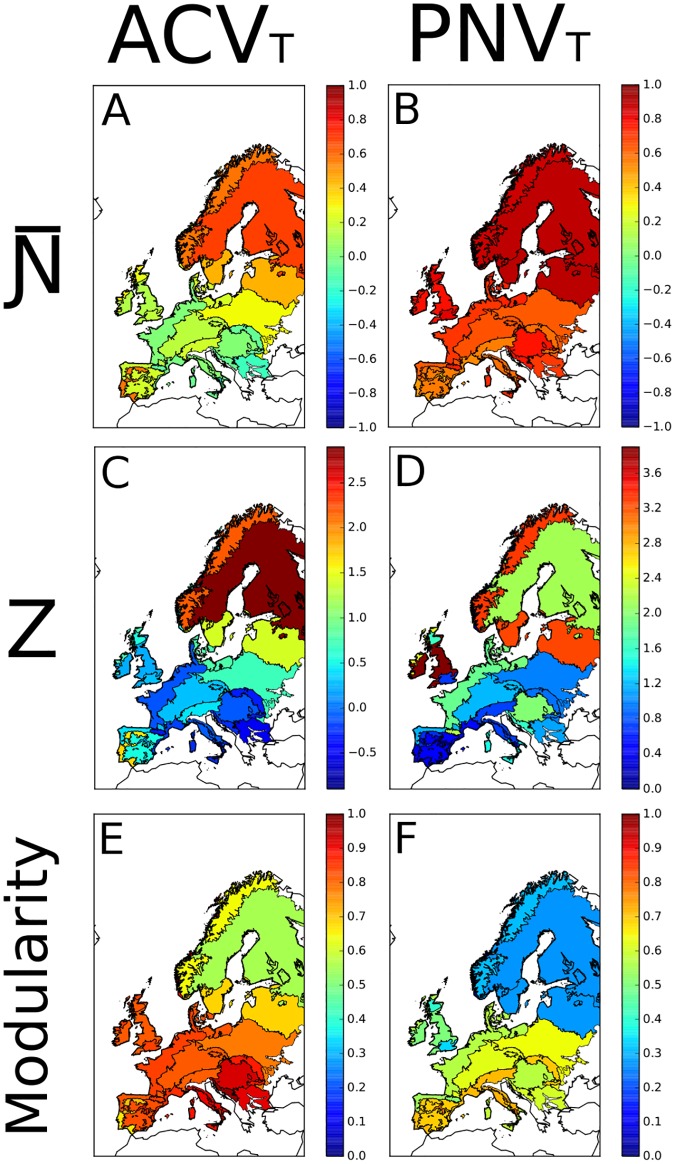
Mapping the structure of ACV_T_ and PNV_T_ across European eco-regions. **$\overset{-}{Ɲ}$**, Z and modularity values computed in all 1° latitude × 1° longitude matrices for both ACV_T_ and PNV_T_, averaged per eco-region.

Mean values of $\overset{-}{Ɲ}$ for PNV_T_ were much higher than those recorded for the ACV_T_, indicating a greater tendency towards nestedness in the potential tree vegetation than in the actual one (Fig 2). Moreover, although we found cases of $\overset{-}{Ɲ}$ = 1 in both ACV_T_ and PNV_T_, only ACV_T_ showed negative $\overset{-}{Ɲ}$ values (Table 1). This is reflected by *Z* values, which were much higher in PNV_T_ than in ACV_T_, with negative values only in the ACV_T_ (Table 1). However, in both PNV_T_ and ACV_T_ significant spatial segregation (i.e. $\overset{-}{Ɲ}$ values close to -1, Z <-2) was extremely rare, while we found significant nestedness (i.e. $\overset{-}{Ɲ}$ values close to 1, Z>2) in many areas (see Figs 3C and 3D and 4C and 4D). In general, PNV_T_ was much more nested than ACV_T_, and particularly at Northern latitudes (Figs 3A and 3B and 4A and 4B).

**Table 1:** Descriptive statistics of $\overset{-}{Ɲ}$, *Z*, and modularity values measured on all the species presence-absence matrices used in the analysis.

	$\overset{-}{Ɲ}$ ACV_T_	$\overset{-}{Ɲ}$ PNV_T_	*Z* ACV_T_	*Z* PNV_T_	*Mod* ACV_T_	*Mod* PNV_T_
Min	-0.733	0.015	-1.868	0.031	0.000	0.000
1st Qu	0.060	0.603	0.113	1.208	0.626	0.000
Median	0.312	0.786	0.667	1.809	0.790	0.566
Mean	0.325	0.757	1.008	2.416	0.730	0.466
3rd Qu	0.628	1.000	1.669	2.730	0.860	0.731
Max	1.000	1.000	21.440	62.877	1.000	1.000

At the scale of eco-regions, $\overset{-}{Ɲ}$ values in PNV_T_ were fairly homogeneous across Europe and, in general, higher than 0.4, i.e. indicating a tendency towards nestedness (Fig 4B). Conversely, the scenario depicted by $\overset{-}{Ɲ}$ values in ACV_T_ is a bit more complex, with higher values in northeastern regions (and in some areas of the Iberian Peninsula) than in the rest of the continent. It is interesting that, as regards PNV_T_, there are several localities having significant *Z* values (i.e. >2) in the United Kingdom, but not in mid-southern Europe (Fig 4C and 4D).

Modularity was more evident for ACV_T_ than for PNV_T_ (Table 1), with most of central and southern Europe having values close to 1 (Fig 3E and 3F). The high modularity values are indicative of the following: (1) species and localities occurred in distinct sets; (2) species of a given set tended to have an overlapping geographic distribution with species of the same set; (3) species of a given set tended to have a disjoint distribution with species of any other set; (4) localities of the same set tended to have similar species composition; and (5) localities belonging to different sets tended to have different species composition. In particular, high modularity of PNV_T_ occurred in central Europe, whereas several localities in Fennoscandia were characterized by very low modularity (Fig 3E and 3F).

Geographical patterns of modularity are even clearer when observed at the level of eco-region. As regards PNV_T_, we found high modularity in mountain regions, and particularly in the Iberian peninsula, and along the Alps, the Apennines and the Carpathians (Fig 4E and 4F). Conversely, we found, on average, low values in Fennoscandia. A strong difference in modularity can be observed between regions above and below the southern coastlines of the North Sea and of the Baltic Sea, with the latter having higher values (especially in the Mediterranean areas) (Fig 4E and 4F).

Actual tree assemblages in localities falling within the few European intact forest landscapes were characterized by higher nestedness and lower modularity in comparison with non-intact forest landscapes (Student’s t-tests with Welch’s correction: *t* = -9.277, *df* = 169.939, *P* < 2.2×10^−16^ for nestedness; *t* = 6.198, *df* = 169.759, *P* < 4.2×10^−9^ for modularity, respectively).

The $\overset{-}{Ɲ}$ measure resulted extremely robust to data availability. Both the values of nestedness and modularity obtained using the whole Euforest dataset were virtually identical to those obtained using a random sample of half the tree occurrence records, with R^2^ values equal, respectively, to 0.91 and 0.78, and both regression lines having a slope close to one and an intercept close to zero (*y* = 0.87*x* + 0.15 for nestedness; *y* = *x* − 0.08 for modularity).

## Discussion

The idea that ecological communities are inherently structured by various processes extends far back in the history of ecological research, at least to Clements' early studies of succession and plant communities as well-organized “super-organisms” [57]. This perspective gained momentum and eventually lead to deterministic views on community structure [14]. Although the idea that communities are structured by species interactions and assembly rules has been challenged by the neutral theory of biodiversity [58, 59], it is reasonable to assume that, given enough time, a certain degree of spatial structure at some scale is likely to emerge in absence of unnatural external disturbance by humans [60, 61]. More cautiously, we could at least assume that natural communities that are frequently disrupted by anthropogenic influences are not as likely to retain structure as are communities that are not disrupted (see, for example, [62]).

Thus, our starting hypothesis was that the continuous and prolonged management of European forests [40] could have led to strong alterations in the natural processes of species interactions and other structuring forces. Although this idea is intuitive, testing it was challenging due to the almost complete lack of information about the level of structure expected in the absence of human disturbance.

Taking advantage of the PNV concept, we were able to fill this knowledge gap, showing that management has set apart the ecological and spatial patterns of contemporary tree communities from those expected in a hypothetical undisturbed scenario. In general, the spatial arrangement of European ACV_T_ resulted highly modular and weakly nested, in clear contrast with that of PNV_T_, that showed high nestedess and low modularity.

The patterns observed in the PNV_T_ are well consistent with the relatively high values of nestedness observed in the ACV_T_ of the few intact forest landscapes (and, in general, at higher latitudes) when compared to those of other European areas. This provides strong support to our idea that mature vegetation should show spatial consistency in species associations, resulting in an overall tendency for positive co-occurrence. Conversely, the scale of our analysis was large enough to rule out the effect of local competition, as confirmed by the fact that we did not detect any patterns of segregation in either ACV_T_ or PNV_T_.

Besides the straightforward idea that nestedness reflects consistency in species associations in mature tree assemblages, our results can also reflect complex biogeographical processes. In a perfectly undisturbed scenario, such as that depicted by PNV_T_, one could expect species assemblages to be driven by colonization and extinction dynamics. These are responsible for species dispersal and replacement along environmental gradients and hence contribute to generating nested patterns. Conversely, nested patterns are likely to be disrupted by human induced biodiversity loss and habitat fragmentation, with the consequent reduction of diversity gradients and, possibly, an increase in modularity. Regardless of their interpretation, however, our results strongly suggest that current European forests are either too ‘young’ to have evolved into a structured system, or, more likely, too much managed to retain the structure predicted by the PNV_T_.

Making comparisons between PNVs and ACVs is often complicated by the fact that the information provided by the first is, in principle, complete, while the latter is not. This can lead to obvious problems in analyzing and interpreting patterns of richness and diversity. However, an important feature of the methodological approach we have used in this study, is its independence from matrix properties [26], which reduces the potential biases due to differences in data availability. As demonstrated by the sensitivity analysis we conducted by using only half the tree occurrence dataset, the presence of a given structural pattern should emerge even if the information is not complete, which ensures that our conclusions are very robust against potential biases due to unequal sampling or data deficiency. This point is key to understanding why using PNVs as null models for ecological structure is fundamentally different (and less problematic) than using them as null models for vegetation types and/or diversity.

The use of PNV as a benchmark was a crucial aspect of our study. The Map of the Natural Vegetation of Europe that we used in this study [49] is the result of more than two decades of joint work of an international team composed by more than 100 vegetation scientists from 31 European countries [63]. Although remarkable, the effort put in compiling the map is by no means a solution to the many problems highlighted by PNV critics [9], and should not be brought into the debate about the general validity of the concept of PNV itself. Our cautious use of PNV illustrates that there is much value in the concept and data generated from PNV databases. We hope that our approach sheds light on what PNVs can tell us, instead of what they cannot.
